# Effect of Ecotype and Starch Isolation Methods on the Physicochemical, Functional, and Structural Properties of Ethiopian Potato (*Plectranthus edulis*) Starch

**DOI:** 10.3390/molecules28217260

**Published:** 2023-10-25

**Authors:** Misikir Milkias, Shimelis Admassu Emire, Workineh Abebe, Felicidad Ronda

**Affiliations:** 1 School of Chemical and Bio-Engineering, Addis Ababa Institute of Technology, Addis Ababa University, King George VI Street, Addis Ababa P.O. Box 385, Ethiopia; misikir.milkias@gmail.com (M.M.); shimelisadmassu@aait.edu.et (S.A.E.); 2 Department of Agriculture and Forestry Engineering, Food Technology, College of Agricultural and Forestry Engineering, University of Valladolid, 47002 Valladolid, Spain; 3 Ethiopian Institute of Agricultural Research, Addis Ababa P.O. Box 2003, Ethiopia

**Keywords:** Ethiopian potato, starch, isolation methods, physico-chemical properties, functional properties

## Abstract

The Ethiopian potato (*Plectranthus edulis*) is an annual tuber crop indigenous to Ethiopia. The crop is underutilized and not much studied despite its high yield of starch, which has a good potential to contribute to the effort in meeting the quickly growing demand for starch. In this study, the effects of the ecotype and isolation methods on the physicochemical, functional, structural, and crystalline properties of starches were evaluated. Starches were isolated from two Ethiopian potato ecotypes (Loffo and Chanqua) using distilled water (DW), 0.01% sodium metabisulphite (SMS), and 1M sodium chloride (NaCl) in the isolation media. The results showed that the lowest starch yield was obtained from Chanqua using DW (97.4%), while the maximum was from Loffo using SMS (99.3%). The L* (lightness) and whiteness values of the starches obtained from Loffo were higher than those of Chanqua starches, with NaCl and SMS extractants yielding the highest values. The bulk density, water activity (a_w_), pH, proximate composition (moisture content, protein, ash, fat, crude fiber, and carbohydrate contents), and techno-functional properties were established. The majority of these parameters varied depending on both the isolation method and the ecotype. The crystallinity pattern of all starches showed B-type diffraction, with differences in diffraction peak intensities between all starches. FTIR tests showed structural changes as a function of the ecotype and isolation procedure used. The Loffo ecotype exhibited considerably better results, and the SMS isolation method was found to be the most effective way to acquire the highest starch quality in most of the characteristics evaluated.

## 1. Introduction

Starch is the abundant carbohydrate resource in plants which serves as a source of carbon and energy in a variety of natural plants [1]. The global industrial starch market is currently expanding quickly due to major factors such as expanding food and beverage, pharmaceutical, and textile industries, improving global economic conditions, rising demand for processed and convenience foods and beverages in developing countries, rising per capita income, and population growth [2]. The food and beverage industry takes the largest portion (>55%) and applies starches or their derivatives as a major ingredient or as an additive to optimize the processing efficiency, product quality, or shelf life [3,4].

The majority of the world’s starch supply comes from corn, potatoes, wheat, cassava, and sweet potatoes [2]. However, these conventional sources are being over-exploited, necessitating the exploration of new botanical sources of starches. Although it is currently receiving little attention, Ethiopian potato (*Plectranthus edulis*) (EP) is one of the extensively grown traditional root crops in Ethiopia, with a high starch content that has a promising potential in contributing to the effort for meeting the quickly growing starch demand [2,5,6]. EP is an annual crop indigenous to Ethiopia which belongs to the *Lamiaceae* family and the genus *plectranthus*. It has various local names such as Dincha Oromo, Wolaita donuwa, Gurage dinch, Hadiya dinch, or Agew dinch. It is grown for its edible tubers [5,7,8] in large hectares in mid and high altitudes of Ethiopia, particularly in the southern, northern, and western parts. The tubers are boiled before consumption [9,10]. EP has a high starch yield that reaches 80.4%, with amylose content ranging from 15 to 23.9% on a dry basis [11], making it an additional rich source of starch that might serve as an alternative to the current starch sources being utilized in the food industry [6].

The application of starches from underutilized tubers like EP requires an in-depth characterization of their physicochemical properties and the selection of a suitable starch extraction method. Studies have shown that variations in the tuber variety or ecotype [12,13,14] and the isolation methods used [14,15,16,17,18] to separate starch from tubers can have an impact on the starch yield and physicochemical, functional, and structural characteristics of starches. The protein, ash, and lipid contents of starches obtained from eight Korean sweet potato varieties varied between 0.01 and 0.28%, 0.10 and 0.12%, and 0.04 and 0.16%, respectively, with the Jeungmi and Shinwhangmi starches having significantly higher protein contents but Shinwhangimi and Shinyulmi having lower lipid contents than other sweet potato varieties [12]. Phogat et al. [14] reported that starch isolated from the potato variety Kufri Chipsona-4 showed the highest yield, water absorption capacity (WAC) (258%), swelling power (SP) (38.4 g/g), and solubility (35.8%) compared to other varieties. Tessema and Admassu [17] reported that the starch yield isolated by the sodium metabisulphite (SMS) (0.075% *w*/*v*) method was significantly the highest (75.56%, db) compared with the sodium chloride (NaCl) and distilled water (DW) methods, with different concentrations from the anchote tuber. According to Xu et al. [18], SMS and sodium hydroxide (NaOH) effectively reduced tissue browning reactions and resulted in the highest starch whiteness value during starch isolation from root tubers of purple, yellow, and white sweet potatoes. Based on our knowledge, however, there is no information available on the effect of different isolation methods and the EP ecotype on the physicochemical, functional, and structural characteristics of EP starch.

In this study, the physicochemical, functional, and structural properties of starch of EP from different EP ecotypes were assessed using the NaCl, SMS, and DW isolation methods. The results of this study will help to choose the most suitable EP ecotype for starch extraction among two of the most mainly produced in Ethiopia and to identify the most appropriate solvent to carry out its isolation process.

## 2. Results and Discussion

### 2.1. Starch Yield 

The starch yield ranged from 97.39% to 99.26% and varied significantly (*p* ≤ 0.05) due to differences in the EP ecotype and starch isolation method (Table 1). The starch yield from the Loffo ecotype was significantly higher than that of Chanqua. The starch yield value obtained from the Loffo isolated by SMS (99.26%) was the highest value, similar to the result reported by Assefa et al. [11]. The SMS and DW media yielded a significantly higher starch isolation efficiency than NaCl, without significant differences between them. This corroborated the finding of Tessema and Admassu [17], who obtained a higher Anchote starch yield from SMS-isolated starch when comparing the effect of different concentrations of SMS and NaCl. SMS, when dissolved in an acidic medium, releases sulfur dioxide. The action of sulfur dioxide is very important because, as a reducing agent, it is capable of breaking the disulfide bonds that wrap matrix protein starch granules to free the starch granules that can help in the separation of starch and increase the amount of starch produced [19].

### 2.2. Physical Properties of EP Starches

The EP ecotype and starch isolation method had a significant effect (*p* ≤ 0.05) on the bulk density (BD), water activity (a_w_), pH, and color of the starches obtained (Table 1). The BD of the isolated EP starches ranged from 0.75 g/cm^3^ (starch isolated from Chanqua with distilled water, CDW) to 0.85 g/cm^3^ (starch isolated from Loffo with NaCl, LNaCl). Starches obtained from Loffo had significantly higher (*p* ≤ 0.05) BDs than those of Chanqua. The variation in BD could be due to the variation in the particle size of starch granules, the compaction profile, and the particle packing arrangement [17,20]. The BD of EP starches also significantly varied with the isolation method, following the order: NaCl (0.84 g/cm^3^) > SMS (0.81 g/cm^3^) > DW (0.76 g/cm^3^). The BD of EP starch was higher than that of the anchote (0.51 g/cm^3^) and potato (0.71 g/cm^3^) starches reported by Tessema and Admassu, regardless of the ecotype and the solvent used in the isolation process [17]. The a_w_ values ranged from 0.38 (LNaCl) to 0.61 (CDW), which is comparable with the a_w_ of the native cassava starch (0.5) reported by Ocieczek et al. [21]. The mean a_w_ of the starch isolates from Loffo had lower values than Chanqua. The mean a_w_ scores of the starch isolated from the three isolation methods also varied significantly (*p* < 0.05), following the order: NaCl (0.40) ˂ SMS (0.51) ˂ DW (0.57). The lower a_w_ value found in starch isolated with NaCl can be attributed to the known decreasing power of this salt on the vapor pressure and water activity of the unbound/available water of the starch, in which it remains dissolved [22]. On the other hand, starch isolates from Loffo had a slightly higher mean pH than those from Chanqua. The isolation methods also affected the mean pH of starch isolates obtained from EP and varied as: DW (5.94) ˂ SMS (7.12) ˂ NaCl (7.34).

### 2.3. Color

Whiteness is one of the physical properties of starch that is important in determining starch quality [18,20]. There were significant differences (*p* ≤ 0.05) between all color parameters due to the EP ecotypes and isolation methods (Table 1). The lightness (L*) and whiteness varied from 95.6 and 94.37, in CDW, to 97.6 and 96.92, in LNaCl, respectively. These whiteness values resulted to be higher than those found in anchote (89.16) and potato starch (93.28) reported by Tessema & Admassu [17]. The L* values measured in EP starches were also above those reported for eight Korean sweet potato varieties (89.7–93.9) [12]. Starch isolation methods significantly (*p* < 0.05) affected L* and whiteness, and the average values of these two parameters respectively varied as: DW (96.3, 95.3) < NaCl (96.5, 95.6) < SMS (97.1, 96.4). The lowest L* and whiteness values were obtained in DW-isolated starches, which could be due to the expected browning reaction during the isolation procedure [12]. The SMS isolation method showed the highest effect in restricting this darkening effect and leading to a significant (*p* ≤ 0.05) increase in L* and whiteness values for the same EP ecotypes. Different studies mentioned that SMS and NaCl could decrease the browning reactions of tissue during starch isolation and, as a result, could give whiter starches [18]. The starch of Loffo EP was found to have higher whiteness than Chanqua and had a significant difference (*p* < 0.05) in all color parameters. Such difference in the whiteness and lightness of the starch extracts could be attributed to the presence of some co-pigmented substances, and variations in their ash and protein contents [12,17,20]. The reddish/greenish (a*/−a*) values ranged from 0.71 (CDW) to −0.38 (LNaCl), whereas the yellowish (b*) values ranged from 1.19 to 3.43. The NaCl and SMS methods had no significant effect on the a* and b* values, while the water isolation method had the lowest (*p* ≤ 0.05) a* and b* values.

### 2.4. Starch Proximate Composition

Except for the fat content, starches isolated from the two EP ecotypes had significant differences (*p* ≤ 0.05) in their moisture, protein, fiber, ash, and carbohydrate contents (Table 2). The isolation method also had a significant (*p* < 0.05) effect on all the compositional parameters measured. 

Moisture content is one of the important factors affecting the physical and functional properties of starch. It has a considerable impact on the flow and other mechanical properties [23,24]. The moisture content varied between 10.23 and 14.80%, where the highest value was obtained in CDW starch and the lowest in LNaCl starch. The EP starch moisture content levels found in this study were comparable with those reported by Hellemans et al. [6] (14.1–17.5%,). In addition, the results were within the range (9.1–17.5%) reported for various tuber starches [2,20] and in the range (10–20%) suggested for commercial starches [25]. The starch isolation method had a significant impact on the moisture content of the resulting starches, and the values varied as follows: NaCl (11.76%) < SMS (13.16%) < DW (13.48%). Based on the ecotype, Loffo EP starch had a lower moisture content (11.42%) than Chanqua starch (14.14%). Tsakama et al. [26] also reported that the moisture content of starches could be significantly affected by the differences in the ecotype.

The fat content of EP starches lied between 0.21% and 0.37%, which was in the range reported by Hellemans et al. (0.15–0.59%) [6] and was near the finding of Assefa et al. (0.21%) [11]. The SMS method-isolated starch had the highest amount of fat content. Similar results (0.50–0.68%) were reported by Julianti et al. [27], who evaluated the effects of the SMS, DW, and citric acid methods on purple-fleshed sweet potato and found that the SMS technique-isolated starches had the highest fat content. Kale et al. [20] reported a 0.09–0.11% fat content in the isolation of sweet potatoes using the NaCl and SMS methods, respectively.

The protein content ranged between 0.65% (CSMS) and 0.95% (LDW), which is more or less in the range reported by Hellemans et al. [6] (0.70–1.76%). The mean protein content of the starch isolated from Chanqua (0.71%) was significantly lower than that of Loffo (0.85%). This shows that the protein content of starches can vary depending on the type of ecotype [12]. The mean protein contents of the EP starches isolated by DW (0.87%) were significantly higher than those isolated by NaCl (0.70%) and SMS (0.76%). NaCl-isolated starch had the lowest (*p* ≤ 0.05) protein content. This could be explained by the fact that NaCl eliminated the protein component by dissolving the starch–protein agglomerates and caused the removal of floating proteins during isolation [16,20]. The protein contents of the EP starches measured were higher than those of the starches isolated from tubers like anchote [2] and purple-fleshed sweet potato [27] 

The values of the fiber contents of the isolated starches ranged between 0.54 (LDW) and 0.89% (CSMS), which were below the values reported by Hellemans et al. [6] (1.2–5.3%). The mean crude fiber and ash in the starches from Chanqua (0.80%, 0.62%) were significantly higher than those of Loffo (0.66%, 0.55%), respectively. The ash content varied between 0.51 and 0.64%, where the highest value was obtained in CSMS starch and the lowest in LDW starch. The variations in the fiber or ash content might be influenced by growth conditions and/or genetic diversity [6]. The isolation method also notably varied the mean ash contents of the resulting starches as NaCl (0.61) > SMS (0.60) > DW (0.55). The reason why EP starches isolated with NaCl had SMS scored relatively higher ash contents could be attributed to the fact that SMS and NaCl contain minerals that are not burned in combustion and increase the accumulation of the mineral content [28]. Kale et al. [20] also mentioned that variations in starches’ ash content could be due to the isolation methods and the degree of homogenization for isolation. The difference in the fiber contents of the starch isolates due to the isolation method was SMS (0.83%) > NaCl (0.73%) > DW (0.64%). The carbohydrate content EP significantly varied between 83.51% (CDW) and 88.12% (LNaCl). The carbohydrate content was influenced by the EP ecotype and isolation method, with the highest values being found in the Loffo ecotype and NaCl isolation method.

### 2.5. X-ray Diffraction of EP Starches

**Figure 1:**
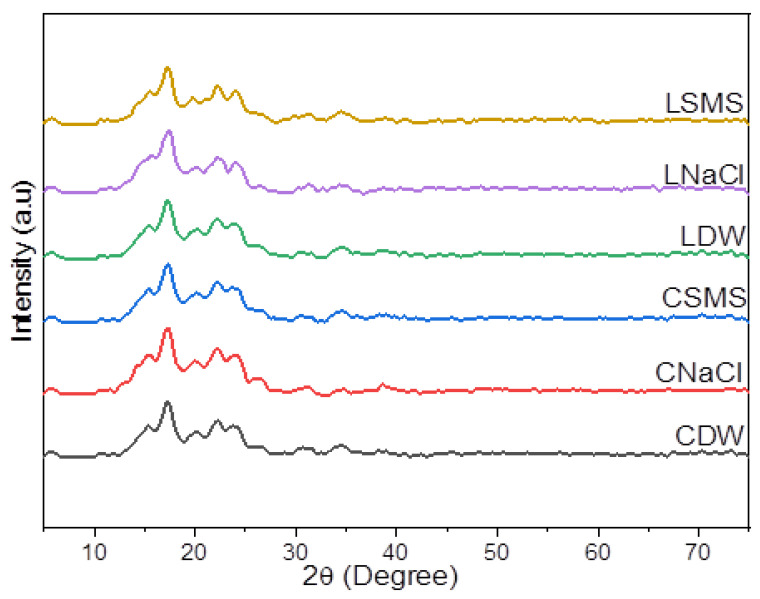
The XRD pattern of starches of Chanqua and Loffo starches. CDW, CNaCl, and CSMS = Distilled water-, Sodium Chloride- and Sodium metabisulfite-isolated Chanqua starch, whereas LDW, LNaCl, and LSMS = Distilled water-, Sodium Chloride- and Sodium metabisulfite-isolated Loffo starch.

The crystallinity pattern of the EP starches showed a B-type XRD pattern (Figure 1), with the characteristic peaks around 5.6°, 15°, 17°, 22°, and 24° 2θ. All starches, however, showed different diffraction peak intensities, which could be attributed to the differences in the ecotype and/or isolation method. According to Wang et al. [29], the environment conditions, especially the growth temperature and moisture, could have a significant impact on the starch’s crystalline structure. Previous researchers found that EP starches exhibited B-type [11] and A- and B-type diffraction [6], whereas potato and cassava starches exhibited B-type and A-type diffraction, respectively [30].

The crystallinity indexes (CI) of EP starches are given in Table 3. The CI values ranged between 30.26 and 38.49%, where the lowest value was recorded for CSMS starch and the highest value was recorded for CSMS. These results showed that the DW, NaCl, and SMS isolation methods had an effect on the CI of starches. The CI values obtained for EP starches in this study were lower than the CI values reported by Tessema & Admassu [17] for anchote (39.15%) and potato (38.60%) and the values stated in Wolde et al. [30] for anchote, cassava, and potato starches, which ranged between 45.7 and 54.4%. Vanier et al. [31] underlined that differences in the crystallinity of starches could be influenced by the crystallite size, the number of crystallites, and the moisture content. They also noted that a decrease in the amorphous area results in an increase in the CI. The relative crystallinity had a significant positive correlation with the moisture content. Wolde et al. [30] also mentioned that crystallinity is directly proportional to the moisture content.

### 2.6. Fourier Transform Infrared (FTIR) Analysis

**Figure 2:**
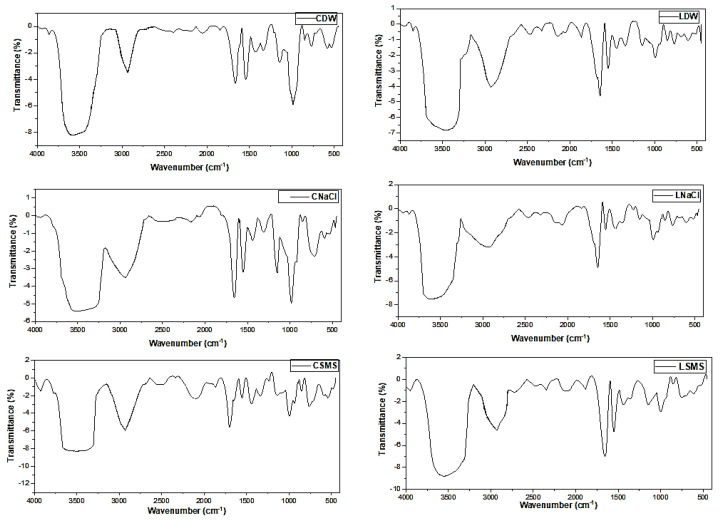
FTIR spectra of the EP starches. CDW, CNaCl, and CSMS = Distilled water-, Sodium Chloride-, and Sodium metabisulfite-isolated Chanqua starch, whereas LDW, LNaCl, and LSMS = Distilled water-, Sodium Chloride-, and Sodium metabisulfite-isolated Loffo starch.

**Figure 3:**
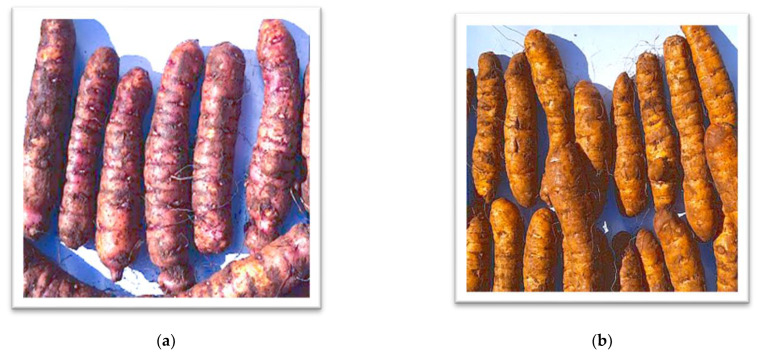
The two types of EP ecotype included in the study: (**a**) Chanqua and (**b**) Loffo.

The FTIR bands obtained for different EP starches are presented in Table 4 and Figure 2. FTIR is the best technique for analyzing and spotting differences in the sample composition, ligand complexation, and structural make-up [32]. All EP starches had broad transmittance bands that fell between 3579 and 3466 cm^−1^, attributed to O-H stretching, and bands between 2929 and 2935 cm^−1^ that show the symmetric/asymmetric stretching modes of the C-H, which agreed with previous reports [33,34,35,36]. There were intense peaks between 1665 and 1647 cm^−1^ (Table 4 and Figure 3), which could be attributed to the C-O bending associated with the OH group and also with H-O-H vibration due to water because the presence of the water molecules bound to the starch tends to create the distinctive peak around 1648 cm^−1^ [36]. The bands that fell between 1362 and 1306 cm^−1^, 1151 and 1141 cm^−1^, and 998 and 983 cm^−1^ were probably due to C-H symmetric bending, C-O-C asymmetric stretching, and C-O stretching, respectively, and there are bands created due to the C-O-C vibration of carbohydrates [37]. The location of the infrared absorption peaks of the six samples had differences in the peak shift to a lower/higher wavenumber or intensity, especially in sensitive regions, because of the effect of the isolation methods and the difference in the EP type (Figure 2).

### 2.7. Functional Properties of EP Starches 

The EP ecotype had significant (*p* ≤ 0.05) effect on the starch water absorption capacity (WAC) and oil absorption capacity (OAC), while the isolation methods had a significant effect on all four of the functional properties evaluated (Table 5). WAC is an important starch processing parameter that is associated with interacting forces inside the starch component, with a higher WAC resulting from lower interaction forces [20,40]. The WAC values ranged from 0.69 g/g (LSMS) to 1.03 g/g (SDW), and closer value ranges were also reported by Hellemans et al. [6]. The variation in WAC could be related to the difference in the ratio of amylose and amylopectin, where a higher amylose content generally results in a lower WAC, while a higher amylopectin content leads to a higher WAC [6]. Starches extracted from the Loffo EP ecotype (0.83 g/g and 0.80 g/g) had a significantly lower mean WAC and OAC than those from Chanqua (0.96 g/g and 1.00 g/g). EP starch isolation by salts (NaCl and SMS) led to a lower starch WAC and OAC in the starches from both EP ecotypes, and depending on the isolation methods, the mean WAC and OAC scores of the starches varied as follows: DW (1.03 g/g) > NaCl (0.85 g/g) > SMS (0.80 g/g) and DW (1.00 g/g) > SMS (0.90 g/g) > NaCl (0.80 g/g), respectively. Comparable WAC and OAC values, (0.88 g/g to 0.95 g/g) and (0.96 g/g to1.15 g/g), were recorded on starches isolated from cassava and sweet potato [41]. The starch swelling power (SP) indicates its capacity to retain water after undergoing heating, cooling, and centrifugation, while water solubility measures the extent to which starch dissolves when it swells in water [18]. The amount of water that is absorbed during gelatinization is what determines the capacity of starch swelling [42]. The effect of the EP ecotype SP and solubility of the isolated starches was not significant. However, the influence of the starch isolation method was important in varying the SP and solubility scores of the isolated starches, respectively, as DW (6.6 g/g) ≥ SMS (6.4 g/g) > NaCl (5.6/g) and DW (6.72%) ≤ SMS (6.53%) < NaCl (7.21%). The starches isolated by NaCl had the lowest SP compared to the others, and this could be attributed to the fact that the salt could impede the swelling and gelatinization of starch [43]. Such significant effect of the isolation media has been reported in starches isolated from root tubers of purple, yellow, and white sweet potatoes [18]. The SP values obtained are negatively correlated (r = −0.78) with the solubility percent, corroborating the earlier findings of Singh Sandhu and Singh [44]. 

## 3. Materials and Methods

### 3.1. Materials

Two types of EP ecotypes, Chanqua and Loffo (Figure 3), were included in this study. The Chanqua and Loffo were collected from Ezzo and Gembelagesha, respectively, in the Southern Nations and Nationalities Peoples Region, Ethiopia, where the EP ecotypes are commonly grown. Analytical grade SMS, NaOH, NaCl, and ethanol were purchased from local chemical markets in Addis Ababa Ethiopia.

### 3.2. Starch Isolation and Yield

Material preparation was conducted following the method described by Hellemnas et al. [6]. Fresh and mature EP tubers were first washed and peeled, and the edible portion of the tuber was cut into smaller pieces/slices; then, the starches were isolated by three different methods depending on the solvent used to perform the extraction. Distilled water (DW), 0.01% sodium metabisulphite (SMS), and 1M sodium chloride (NaCl) were used as solvents following the methods described in Kale et al. [20], Surendra Babu & Parimalavalli [16], and Xu et al. [18], with some modifications. The slices of EP tubers were blended with the different solvents at a ratio of 1:10 (*v*/*v*) using a laboratory-grade blender (Universal fritter QS806, Chana, IL, USA) until a smooth slurry was formed. Then, the slurries were filtered with double-layered cheesecloth and centrifuged (TGL-16, Sichuan Shoke, Leshan, China) for 20 min at 5000× *g* and 20 °C. The supernatant was decanted, and the starch settled at the bottom of the centrifuge tube was washed with distilled water to remove impurities. Finally, the starch was allowed to dry overnight at room temperature, ground with a mortar and pestle into fine powder, and packed in polyethylene bags for further investigations.

The starch yield was determined, as described in Awolu et al. [45], by calculating the starch obtained as the percentage ratio of starch recovered after isolation to the EP tuber sample utilized.

### 3.3. Starch Bulk Density

The starch bulk density (BD) was determined following the method described in Stasiak et al. [46]. A total of 20 g of the starch sample was gently loaded into a 100 mL graduated cylinder. The measured volume was used to calculate the bulk density according to the mass/volume ratio.

### 3.4. Color

The color measurements of the starches were carried out using a Color Measuring System (Hunter Lab colorimeter, Minolta). L*, a*, and b* coordinates were obtained with the D65 standard illuminant and 10° standard observer. The whiteness of the starches was determined as per the method described by Kale et al. [20], where the whiteness of starch was calculated using Equation (1).

Whiteness = 100 − [(100 − L*)^2^ + a*^2^ + b*^2^]^½^ (1)

where: L* = lightness, b* = yellowness, and a*= greenness.

### 3.5. Starch Proximate Compositions

The moisture, protein, fat, crude fiber, and total ash contents of the extracted EP starches were determined according to method given by AACC, 2001 [47]. The carbohydrate content was determined by subtracting the sum of the percentages of moisture, ash, protein, and lipid contents from 100 [48].

### 3.6. Functional Properties

The water absorption capacity (WAC) was measured according to the method described in Surendra Babu & Parimalavalli [16]. A total of 1 g of the EP starch sample was weighed into a pre-weighed centrifuge tube, and 10 mL of distilled water was added. The mixture was allowed to stand for 30 min and centrifuged at 3500× *g* for 15 min, and the supernatant was discarded. The tube was allowed to drain for 10 min at a 45° angle. Subsequently, the sample tube was weighed, and the gain in weight was used to calculate the WAC, which was expressed as g H_2_O/g starch.

The oil absorption capacity (OAC) was determined according to the method used by Bikila et al. [49]. A total of 1 g of the starch sample (m_1_) was immersed in 10 mL of soybean oil at room temperature, shaken to mix well, and then left for 30 min to reach the maximal absorption. Then, the mixture was centrifuged at 1811× *g* for 30 min, and the clear supernatant was decanted. Additionally, the sediment was weighed (m_2_). Finally, the OAC was calculated using Equation (2):

(2) $$\mathrm{OAC}\left(\frac{g}{g}\right)=\frac{m_{2}-m_{1}}{m_{1}}$$

The starch swelling power (SP) and solubility were studied by the method used by Shimels et al. [50] and Kaur et al. [51], with a slight modification. A total of 1 g (dry basis) of the EP starch sample was mixed with 10 mL of distilled water in a centrifuge tube and heated in a water bath at 90 °C for 30 min. After heating, the suspension was centrifuged at 2200× *g* for 15 min. The supernatant was drawn off by suction and dried in an oven at 120 °C for 4 h. The solutes that remained after evaporation were weighed, and the solubility, expressed as a percentage, was calculated using Equation (3). The SP was calculated as the weight of the sediment paste per gram of starch, as stated in Equation (4).

(3) $$\mathrm{Solublity}\;\left(\%\right)=\frac{\mathrm{Weight}\;\mathrm{of}\;\mathrm{soluble}\;\mathrm{starch}\;\left(g\right)}{\mathrm{Weight}\;\mathrm{of}\;\mathrm{starch}\;\left(g\right)}\times 100$$

(4) $$\mathrm{SP}\left(g/g\right)=\frac{\mathrm{Weight}\;\mathrm{of}\;\mathrm{sedment}\;\mathrm{paste}\;\left(g\right)}{\mathrm{Weight}\;\mathrm{of}\;\mathrm{starch}\;\left(g\right)-\mathrm{Weight}\;\mathrm{of}\;\mathrm{soluble}\;\mathrm{starch}\;\left(g\right)}\times 100$$

### 3.7. Crystalline Structure and Average Crystallite Size of EP Starches 

The crystalline structure of the starches was analyzed as described by Singh et al. [39] using an X-ray powder diffractometer (XRD-7000, Drawel, Drawel scientific instrument Co., Ltd., Shangai, China) equipped with a copper tube operating at 35 kV (25 mA), with CuKα radiation of a 0.154 nm wavelength. The samples were scanned from 5° to 75 (2θ) at a rate of 10°/min.

The crystallinity index (CI) was calculated as described by Singh et al. [39] from the ratio of the integrated area of all crystalline peaks to the total (crystalline plus amorphous) integrated area under the XRD peaks (Equation (5)).

(5) $$\mathrm{Crystallinity}\;\mathrm{Index}=\frac{\mathrm{Area}\;\mathrm{under}\;\mathrm{crystalline}\;\mathrm{portion}\;}{\mathrm{Total}\;\mathrm{area}}\times 100$$

### 3.8. Fourier Transform Infrared Analysis

The FTIR analysis was conducted following the procedure used by Awolu et al. [45]. The starch samples were treated with equal quantities of potassium bromide (KBr) salt, and each sample was pressed in KBr Salt Plates and transferred to the Fourier transform infrared spectrometer (Perkin-Elmer spectrometer, Liantrisant, UK) with Spectrum 10^TM^ software, V.10.4.3. The range of the scanning wavenumber was 4000–450 cm^−1^.

### 3.9. Data Analysis

Analysis of Variance (ANOVA) was performed using the statistical software OriginPro 2019b, SAS version 9.0, and MS Excel 2016. Tukey’s multiple comparison test was used to compare the individual difference in the physicochemical properties of the starches. A 95% confidence interval (*p* ≤ 0.05) was considered.

## 4. Conclusions and Recommendation

In this study, two EP (Chanqua and Loffo ) ecotypes and three different starch isolation methods were evaluated to determine their effect on the physicochemical, functional, and structural properties of EP starches. The isolation method used had a significant (*p* < 0.05) impact on the yield, physicochemical properties (bulk density, a_w_, pH, color, moisture, protein, ash, and fiber), and functional properties (WAC, OAC, SP, and solubility). Similarly, the ecotype had a significant impact on the characteristics, with the exception of fat, SP, and solubility. The XRD pattern of all starches showed B-type diffraction, with a difference in the diffraction peak intensities between all starches, which could be attributed to the differences in the ecotype and/or isolation methods. The results from the FTIR tests confirmed differences in the location (wavenumber) and intensity of the infrared absorption peaks of all the samples, especially in sensitive regions, depending on the ecotype and isolation method of EP starches. Between the two EP ecotypes, Loffo exhibited considerably better results in terms of the starch yield, color/whiteness, and some other physicochemical properties. The SMS isolation method was the most effective in acquiring better EP starch characteristics in most of the parameters evaluated. 

## Figures and Tables

**Table 1:** The EP starches yield and physical properties.

Sample	Starch Yield % (db)	Bulk Density (g/cm^3^)	Water Activity (a_w_)	pH	Color			
					L*	a*	b*	Whiteness
CDW	98.07 ± 0.07 ^ab^	0.746 ± 0.011 ^d^	0.605 ± 0.001 ^a^	5.82 ± 0.01 ^e^	95.6 ± 0.2 ^c^	0.70 ± 0.09 ^a^	3.4 ± 0.3 ^a^	94.4 ± 0.3 ^d^
CNaCl	97.39 ± 0.53 ^b^	0.832 ± 0.001 ^a^	0.421 ± 0.002 ^e^	7.29 ± 0.01 ^b^	96.6 ± 0.2 ^b^	−0.07 ± 0.04 ^c^	2.2 ± 0.2 ^c^	95.8 ± 0.4 ^b^
CSMS	98.41 ± 0.55 ^ab^	0.806 ± 0.001 ^b^	0.572 ± 0.003 ^b^	7.23 ± 0.03 ^b^	96.5 ± 0.1 ^b^	0.18 ± 0.05 ^b^	2.6 ± 0.1 ^c^	95.7 ± 0.1 ^b^
LDW	99.11 ± 0.09 ^a^	0.781 ± 0.001 ^c^	0.530 ± 0.003 ^c^	6.07 ± 0.12 ^d^	95.9 ± 0.2 ^c^	0.11 ± 0.06 ^d^	3.1 ± 0.1 ^b^	94.9 ± 0.2 ^c^
LNaCl	98.65 ± 0.70 ^a^	0.852 ± 0.018 ^a^	0.380 ± 0.011 ^f^	7.39 ± 0.01 ^a^	97.6 ± 0.2 ^a^	−0.38 ± 0.14 ^d^	1.2 ± 0.1 ^d^	96.9 ± 0.2 ^a^
LSMS	99.26 ± 0.22 ^a^	0.806 ± 0.002 ^b^	0.441 ± 0.001 ^d^	7.01 ± 0.03 ^c^	97.4 ± 0.3 ^a^	−0.25 ± 0.15 ^b^	1.9 ± 0.2 ^d^	96.8 ± 0.2 ^a^
Analysis of variance and significance (*p*-values)								
Ecotype (A)	*	*	*	*	*	*	*	*
Method (B)	*	*	*	*	*	*	*	*
Interaction (A × B)	*	*	*	*	*	*	*	*

Where CDW, CNaCl, and CSMS, respectively, represent Chanqua starch isolated by Distilled water, Sodium Chloride, and Sodium metabisulfite; LDW, LNaCl, and LSMS, respectively, represent Loffo starch isolated by Distilled water, Sodium Chloride, and Sodium metabisulfite. L* = lightness value, 100 = white and 0 = black, a* = red (+)/green (−) and b* = yellow (+)/blue (−). Data are expressed as the mean ± standard deviations of triplicate assays and seven measurements for color. Different superscripts in the same column indicate statistically significant differences (*p* ≤ 0.05); * = Significant (*p* < 0.05)

**Table 2:** Chemical composition of *EP* starches (in % dry basis, except in moisture content).

Starch	Moisture	Fat	Protein	Crude Fiber	Ash	Carbohydrate
CDW	14.80 ± 0.22 ^a^	0.31 ± 0.03 ^ab^	0.78 ± 0.15 ^ab^	0.199 ± 0.000 ^c^	0.64 ± 0.19 ^a^	83.46 ± 0.17 ^d^
CNaCl	13.28 ± 0.08 ^b^	0.21 ± 0.01 ^c^	0.68 ± 0.01 ^b^	0.305 ± 0.009 ^b^	0.63 ± 0.03 ^a^	85.19 ± 0.19 ^c^
CSMS	14.44 ± 0.51 ^a^	0.37 ± 0.19 ^a^	0.65 ± 0.06 ^b^	0.301 ± 0.003 ^b^	0.58 ± 0.21 ^ab^	83.93 ± 0.46 ^d^
LDW	12.15 ± 0.10 ^c^	0.26 ± 0.03 ^bc^	0.95 ± 0.03 ^a^	0.202 ± 0.005 ^c^	0.51 ± 0.01 ^c^	86.11 ± 0.11 ^b^
LNaCl	10.23 ± 0.10 ^d^	0.32 ± 0.01 ^ab^	0.72 ± 0.01 ^b^	0.298 ± 0.000 ^b^	0.59 ± 0.03 ^ab^	88.12 ± 0.08 ^a^
LSMS	11.88 ± 0.11 ^c^	0.34 ± 0.01 ^a^	0.87 ± 0.09 ^ab^	0.398 ± 0.001 ^a^	0.55 ± 0.01 ^bc^	86.35 ± 0.18 ^b^
Analysis of variance and significance (*p*-values)						
Ecotype (A)	*	NS	*	*	*	*
Method (B)	*	*	*	*	*	*
Interaction (A × B)	*	*	*	*	*	*

CDW, CNaCl, and CSMS = Distilled water-, Sodium Chloride-, and Sodium metabisulfite-isolated Chanqua starch, whereas LDW, LNaCl, and LSMS = Distilled water-, Sodium Chloride- and Sodium metabisulfite-isolated Loffo starch. Data are expressed as the mean ± standard deviations of triplicate assays. Different superscripts in the same column indicate statistically significant differences (*p* < 0.05); * = significant (*p* < 0.05) and NS = not significant.

**Table 3:** Characteristic peaks, CI, and average crystallite/grain size of the EP starches.

EP Starch	Major Peaks (2θ)					CI (%)
	1	2	3	4	6	
CDW	5.65	15.37	17.29	22.21	23.71	38.49
CNaCl	5.68	15.46	17.29	22.21	24.07	37.59
CSMS	5.59	15.67	17.53	22.51	24.31	30.26
LDW	5.66	15.41	17.29	22.31	23.73	33.72
LNaCl	5.69	15.71	17.41	22.24	23.95	35.25
LSMS	5.60	15.52	17.29	22.24	24.01	31.74

CDW, CNaCl, and CSMS = Distilled water-, Sodium Chloride-, and Sodium metabisulfite-isolated Chanqua starch, whereas LDW, LNaCl, and LSMS = Distilled water-, Sodium Chloride-, and Sodium metabisulfite-isolated Loffo starch; CI = crystallinity index.

**Table 4:** FTIR Frequencies region and absorbing features.

Chanqua			Loffo			Frequencies Region and Absorbing Features of Functional Groups Obtained from the Literature		References
DW	NaCl	SMS	DW	NaCl	SMS			
3578	3529	3500	3466	3586	3556	3600–3300, 3750–2800 cm^−1^	O-H stretching	[37]
2933	2935	2930	2931	2930	2929	3000–2900, 2930	C-H stretching	
1665	1654	1649	1655	1647	1660	1637	C-O bending associated with OH	[32]
1430	1551	1558	1547	1552	1552	1458	CH_2_ symmetric deformation	
1423	1442	1441	1450	1435	1435	1415	CH_2_ symmetric scissoring	[33]
1333	1306	1335	1348	1351	1362	1385–1375	C-H symmetric bending	
1144	1148	1140	1147	1151	1141	1149, 1300–1000 cm^−1^	C-O-C asymmetric stretching	[34]
985	983	995	997	993	998	1200–800	C-O stretching	
852	921	930	936	936	852	920	C-O-C vibration of carbohydrate	[35]
765	854	853	856	853	760	856		[38]
	711	761	769	765		758		[39]

DW, NaCl, and SMS represent starches extracted by Distilled water, Sodium Chloride, and Sodium metabisulphite, respectivly.

**Table 5:** Functional properties of EP starches.

EP Starch	WAC (g/g)	OAC (ml/g)	SP (g/g)	Solubility (%)
CDW	1.039 ± 0.005 ^a^	1.196 ± 0.001 ^a^	6.68 ± 0.12 ^a^	6.83 ± 0.09 ^b^
CNaCl	0.932 ± 0.009 ^b^	0.796 ± 0.001 ^c^	5.36 ± 0.15 ^b^	7.30 ± 0.05 ^a^
CSMS	0.908 ± 0.020 ^b^	0.995 ± 0.001 ^b^	6.52 ± 0.33 ^a^	6.22 ± 0.05 ^c^
LDW	1.028 ± 0.008 ^a^	0.796 ± 0.000 ^c^	6.53 ± 0.09 ^a^	6.20 ± 0.15 ^c^
LNaCl	0.785 ± 0.000 ^c^	0.798 ± 0.001 ^c^	5.77 ± 0.08 ^b^	7.12 ± 0.04 ^a^
LSMS	0.691 ± 0.002 ^d^	0.798 ± 0.001 ^c^	6.28 ± 0.01 ^a^	6.83 ± 0.05 ^b^
Analysis of variance and significance (*p*-values)				
Ecotype (A)	*	*	NS	NS
Methods (B)	*	*	*	*
Interaction (A × B)	*	*	*	*

CDW, CNaCl, and CSMS = Distilled water-, Sodium Chloride-, and Sodium metabisulfite-isolated Chanqua starch, whereas LDW, LNaCl, and LSMS = Distilled water-, Sodium Chloride-, and Sodium metabisulfite-isolated Loffo starch. WAC = Water absorption capacity, OAC = Oil absorption capacity, and SP = Swelling power. Data are expressed as the mean ± standard deviations of triplicate assays. Different superscripts in the same column indicate statistically significant differences (*p* < 0.05); * = significant (*p* < 0.05) and NS = not significant.

## Data Availability

Authors will avail data upon request.
